# Renal epithelioid angiomyolipoma with a negative premelanosome marker immunoprofile: a case report and review of the literature

**DOI:** 10.1186/1752-1947-7-118

**Published:** 2013-04-29

**Authors:** Samantha E Hohensee, Francisco G La Rosa, Petra Homer, Thomas Suby-Long, Shandra Wilson, Scott M Lucia, Kenneth A Iczkowski

**Affiliations:** 1 Department of Pathology, University of Colorado, Anschutz Medical Campus, 12800 East 19th Avenue Mail Stop 8104, Aurora, CO 80045, USA; 2 Department of Radiology, University of Colorado, Anschutz Medical Campus, Aurora, CO 80045 USA; 3 Department of Urologic Oncology, University of Colorado, Anschutz Medical Campus, Aurora, CO 80045, USA

## Abstract

**Introduction:**

The rare variant of renal epithelioid/pleomorphic angiomyolipoma has been reported in approximately 120 cases. One of the most important characteristics to differentiate these tumors from other renal cell neoplasms is their typical reactivity to premelanosome antigens. If such a tumor does not stain for HMB-45 or Melan-A, a specific diagnosis of epithelioid pleomorphic angiomyolipoma cannot be made with certainty.

**Case presentation:**

We present here what is, to the best of our knowledge, the first case of epithelioid/pleomorphic angiomyolipoma of the kidney in a 50-year-old Caucasian man with no history of tuberous sclerosis, and with a tumor marker profile negative for several premelanosome antigens. The tumor was composed of sheets of pleomorphic, round to polygonal epithelioid cells with prominent eosinophilic cytoplasm, large nuclei, many multinucleated, and very prominent nucleoli. There were prominent vessels and rare interspersed smooth muscle fibers, but adipocytes were not identified. A tumor marker profile showed tumor cell reactivity for CD68, calponin and focally for CD10. Intervening smooth muscle was reactive with smooth muscle actin. The tumor lacked reactivity for melanin-associated antigens HMB-45 and Melan-A, and for CD31, pan-cytokeratin (AE1/3) and desmin. Electron microscopic examination of tumor cells confirmed the presence of premelanosome-like granules.

**Conclusions:**

Based on the characteristic microscopic appearance of this tumor, and its overall tumor marker profile, we concluded this was a renal epithelioid/pleomorphic angiomyolipoma with a negative premelanosome antigen phenotype.

## Introduction

Angiomyolipomas (AML) are members of a family of tumors derived from mesenchymal, peri-vascular epithelioid cells, normally clustered around blood vessels, and usually named PEComas (for ‘perivascular epithelioid cell tumors’). Renal AML contain varying amounts of three major histological components: blood vessels, smooth muscle, and adipose tissue. AML comprises 2.0 percent to 6.4 percent of all renal tumors and is most commonly benign in behavior [1]. AML can occur as an isolated lesion or as part of the tuberous sclerosis complex (45 percent of cases).

Rare cases of AML, composed of a prominent epithelioid component, with spindle and giant cells, containing none or a minimal amount of adipose tissue, have been reported as the epithelioid variant of angiomyolipoma (EAML) [2]. Up to one-third of these variants show a more aggressive or malignant clinical behavior, yet their overall prognosis has not been clearly defined [1,3]. The epithelioid cells in EAML can be quite pleomorphic and the appearance of a carcinoma-like pattern is frequently observed. Additional focal necrosis, hemorrhage, increased mitotic activity and larger sizes are usually correlated with more aggressive, malignant behavior. These features might lead to an erroneous diagnosis of high-grade renal cell carcinoma, particularly since intra-tumoral fat can occur in these tumors [4]. In the approximately 120 reported cases of renal EAML, the presence of premelanosome antigens (PMA) has been used as one of the main diagnostic landmarks [1,3]. This is in addition to the expression of some other important markers such as calponin and CD68, and the lack of expression of distinct epithelial cell markers such as cytokeratin AE1/AE3, high molecular weight cytokeratin and CAM5.2 [5]. Thus, if such a tumor does not stain for HMB-45 or Melan-A, a specific diagnosis of EAML cannot be made with certainty. We report here a rare case of a 7cm renal tumor in a 50-year-old man, which we classify as a renal EAML despite its negative PMA phenotype.

## Case presentation

Our patient was a 50-year-old Caucasian man with a medical history significant for hypertension, obesity, and nephrolithiasis, and no history of tuberous sclerosis, who presented to our facility with a five-day history of fever, chills, night sweats and lethargy. He had intentionally lost just over 9kg (20 pounds) while taking a product called ‘Healthy Trim’ during the previous month, but had stopped taking it approximately one week prior to presentation. Initial laboratory test results included only a mild normochromic normocytic anemia and an increased erythrosedimentation rate of 86mm/hour. A chest X-ray showed clear lungs with no evidence of consolidation, pleural effusion or pneumothorax; his heart was of normal size.

**Figure 1:**
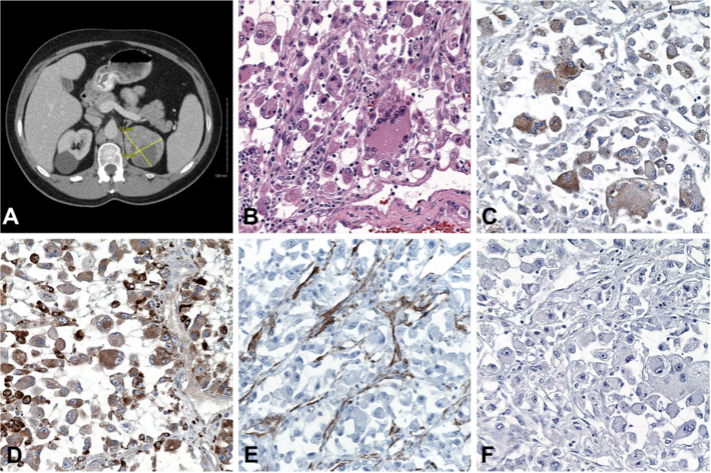
**(A) Computed tomography scan showing a heterogeneous hypo-dense mass in the left kidney measuring 73×66×65mm across its maximal dimensions (crossing yellow bars).** A 35mm simple cyst is present in the right kidney. (**B**) Microscopic image from hematoxylin and eosin sections of the epithelioid variant of angiomyolipoma tumor showing sheets of pleomorphic, round to polygonal epithelioid cells, with prominent eosinophilic cytoplasm, large nuclei, many of them multinucleated, and with very prominent nucleoli; some interspersed smooth muscle fibers were present but no adipocytes were identified (40× objective). Microscopic images from the renal epithelioid angiomyolipoma with immunoperoxidase stainings showing positive reactivity for calponin (**C**), CD68 (**D**), smooth muscle actin (**E**), and negative reactivity for HMB45 (**F**) (all at 40× objective).

His symptoms continued and approximately two weeks later a chest, abdomen and pelvis computed tomography (CT) scan was performed. This imaging study showed a solid heterogeneous mass (73×66×65mm) involving the parenchyma of the upper pole of the left kidney (Figure 1A). It extended exophytically from the superior and posterior-medial aspects of the kidney. No renal vein invasion or regional lymph node enlargement was detected. The right kidney showed a 35mm simple, homogeneous cystic formation with no evidence of solid component; no other solid renal masses were identified.

Our patient underwent a hand-assisted laparoscopic left nephrectomy with adrenalectomy without complications. Hand-assisted laparoscopic nephrectomies are performed per our institution’s protocol in all cases of renal tumors to avoid morcellation and to maintain the integrity of the specimen. After surgery, a gross surgical pathology examination revealed a 19×8×6.5cm left kidney with a 3.5×1×1cm adrenal gland. The external surface of the specimen was inked black and the specimen was then bi-valved, revealing a 7.5×5.1×4.0cm pinkish/tan, extremely friable and necrotic mass located in the superior pole of the kidney. The mass appeared well circumscribed with pushing borders that displaced, but appeared to not invade, into the superior peri-renal adipose tissue. The inferior portion of the mass involved the renal pelvis. The mass extended to 0.1cm from the superior margin and 4.5cm from the renal vein margin; no tumor thrombus in the renal vein was present. The uninvolved renal tissue showed a well-defined corticomedullary junction with an average cortical thickness of 1.2cm. There was a single calcified, 1.0×0.5×0.5cm renal calculus in the inferior pole. The adrenal gland was grossly unremarkable with no lesions identified. Representative sections were submitted for histological preparations.

Histologically, sections of the mass showed round pleomorphic and occasionally multinucleated tumor cells with eosinophilic cytoplasm, macronucleoli, and rare mitotic figures; moderate interspersed smooth muscle fibers were observed, but adipocytes were not identified (Figure 1B). There was prominent vasculature, focal necrosis and hemorrhage; a microfocus of tumor invasion into the peri-renal adipose tissue was present with no invasion beyond Gerota’s fascia. No regional lymph nodes were identified. The Pathologic Staging pTNM of the tumor was pT3a, Nx, Mx (Stage III).

Immunoperoxidase stainings showed tumor cells with uniform cytoplasmic reactivity for vimentin, calponin (Figure 1C), and CD68 (Figure 1D). The tumor cells had patchy reactivity for CD10 and epithelial membrane antigen (EMA). Smooth muscle actin (SMA) stained only the intervening smooth muscle slips and small round capillaries (Figure 1E). Immunostaining for HMB-45 (Figure 1F) and Melan-A were performed on two separate areas of the tumor and using two different chromogens, diaminobenzidine (DBA) and alkaline phosphatase (AP), and all showed negative staining of the tumor cells. Negative stainings were also observed for renal cell carcinoma (RCC) antigen, cytokeratin AE1/AE3, cytokeratin 7, high molecular weight cytokeratin (MA903), CAM5.2, CD1, CD31, CD117, S100, microphthalmia-associated transcription factor (MiTF), tyrosinase (DBA and AP), and desmin (only blood vessels were positive). Staining for Ki-67 (MIB-1) was performed to evaluate the proliferative activity of the tumor, which showed positive nuclear staining in an average of approximately 30 percent of the tumor cells. The positive and negative controls reacted appropriately for each staining.

**Figure 2:**
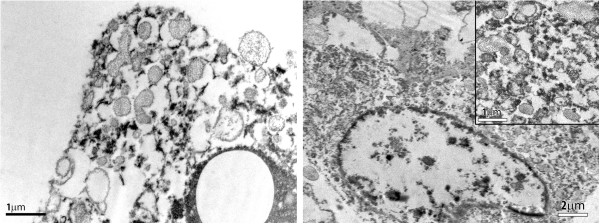
**Electron microscopic image of renal epithelioid angiomyolipoma cells bearing cytoplasmic organelles consisting of premelanosomes, similar to those reported previously **[6]**.**

Tissue retrieved from a paraffin block was subjected to electron microscopic examination in an attempt to demonstrate the premelanosome-like granules that are usually present in epithelioid angiomyolipoma cells. While the paraffin sample used for this study provided suboptimal tissue fixation, we were able to identify tumor cells with finely granular organelles with a substructural periodicity resembling that of premelanosomes (Figure 2) [6].

In the 26 months of clinical follow-up data available at the time of submission of this report, four chest/abdomen/pelvis CT scans were performed, none of which demonstrated evidence of local recurrence or metastatic disease. Our patient did not receive chemotherapy or radiation therapy.

## Discussion

Renal EAMLs are rare neoplasms with approximately 120 cases reported in the literature [3]. After the discovery of AML’s association with HMB-45 in 1991, reports of EAMLs began to appear in the literature [1-5]. In 2009, Tsai *et al*. published five cases from their practice within the previous 10 years and summarized 17 other cases from the recent literature [5]. Similar to other case reports, Tsai *et al*. found that approximately half of EAML cases are associated with the tuberous sclerosis complex, all contained minimal to no fat and all demonstrated a positive reaction to PMA [5].

Distant metastases to the lungs from renal EAMLs were first described in 1991 by Ferry *et al*. [7]. In that report, the observed distant metastases were biopsy proven and ultimately fatal. In a recent series review of 33 pure EAML cases, Nese *et al*. found local recurrence in 17 percent, metastasis in almost 50 percent, and death due to disease in approximately one-third of cases [3]. Conversely, Aydin *et al*. followed 15 patients with EAML for a mean of 5.1 years and none had local recurrence or metastases [1]. Many of these apparently benign cases had atypical histologic features such as coagulative tumor necrosis, nuclear atypia and mitotic activity, thus highlighting the fact that reliable morphologic criteria for malignancy in EAMLs have not been clearly identified [1].

Due to their varying appearances and pleomorphism, renal EAMLs can pose diagnostic difficulty. Eble *et al*. reported five EAML tumors in patients from 20 to 48 years of age and observed that their different microscopic features resembled other benign or malignant tumors [8]. Spindle-shaped smooth muscle cells, epithelioid cells and abundant atypism and pleomorphism mimicked leiomyomas, renal cell carcinomas and leiomyosarcomas [8], with the additional and paradoxical association of lack of adipose tissue. Additionally, gross extension into peri-renal tissue and microscopic entrapment of normal renal tissue caused suspicion of a more invasive process [8].

Immunohistochemistry was valuable in sorting through the sometimes lengthy differential diagnoses. Compared to renal cell carcinomas, renal EAML tumors are distinctively negative for cytokeratin and other epithelial markers, and positive for PMA, CD68 and calponin. Even though a few angiolipomas and other PEComas arising in non-renal locations (that is, liver, lungs, and so on) have been reported to have patchy or negative reactivity for melanosome markers, recent reviews consider that 100 percent of renal EAMLs are positive for either HMB-45 or Melan-A [5,9-12]. Thus, if they do not stain for HMB-45 or Melan-A, a specific diagnosis of EAML cannot be made with certainty.

In our patient’s case, we observed tumor cells with morphological features of EAML both by light and electron microscopic examination. In addition, tumor cells showed negative staining for all but one of the conventional renal carcinoma epithelial markers (EMA). Even though the presence of EMA expression in our patient’s case is quite puzzling; it is generally accepted that aberrant antigenic expression of non-organ related antigenic markers is a fairly common feature in tumor biology. EMA expression has been reported in many non-epithelial tumors, even in some lymphomas.

To explain the lack of PMA expression in our patient’s case, we could use a similar rationale as discussed above for the aberrant antigenic expression. It is tempting to speculate that specific mutation(s) of the genes expressing premelanosome epitopes are missing in our patient’s case, although specific molecular genetic studies have not been performed for confirmation. PMA negative expression has been described even in melanoma cases, and electron microscopic studies need to be performed to confirm the presence of premelanosome structures, similar to other EAML cases reported earlier [6]. Premelanosome-like structures seen in EAML are not melanized; they usually have the capacity to produce tyrosinase in approximately 50 percent of cases, which explains their granular appearance, but not melanin. Thus, the possibility of unusual immunophenotypic expression should always be considered when the morphologic and molecular manifestations of a tumor are discrepant.

An extended review of the literature has not revealed a single report of a renal EAML without positivity for at least one premelanosome marker. Zavala-Pompa *et al*. studied 21 classic angiomyolipomas, in which Melan-A was positive in 18 cases (86 percent), HMB-45 in 16 cases (77 percent), and MiTF in 76 percent. Among four EAMLs, three were positive for Melan-A, three were positive for MiTF, but all four were positive for HMB-45 [13]. Likewise, all other case reports of renal EAML in which these markers were studied had documented reactivity for Melan-A and/or HMB-45 [3,5,7,8,10,11].

Management of EAMLs has not been extensively studied. Surgical resection of these tumors frequently occurs because they are radiologically mistaken for renal cell carcinomas and confirmatory biopsies are many times not preformed. Mutations in tuberous sclerosis genes result in a constitutive activation of the mammalian target of rapamycin (mTOR) and the drug sirolimus (also known as rapamycin) suppresses such mTOR signaling. Thus, sirolimus has reportedly been successfully used in AMLs [14], as well as in some [15] but not all [16] cases of other tumors of the PEComa family.

## Conclusions

Based on the characteristic microscopic appearance of this tumor, its overall tumor marker profile and identification of premelanosome structures by electron microscopy, we conclude that it was a renal EAML with lack of reactivity for premelanosome markers, a finding that makes this tumor different from all previously reported cases.

## Consent

Written informed consent was obtained from our patient for publication of this case report and any accompanying images. A copy of the written consent is available for review by the Editor-in-Chief of this journal.

## Abbreviations

AML: Angiomyolipomas; EAML: Epithelioid angiomyolipoma; PMA: Premelanosome antigens.

## Competing interests

The authors declare that they have no competing interests.

## Authors’ contributions

SEH, FGLR and KAI were the major contributors in writing the manuscript. TS-L performed the radiological studies. SW performed the surgical procedure. SEH, FGLR and KAI performed the original histological examination of the renal tumor, interpreted and diagnosed the pathology findings. FGLR prepared the figures, did the literature review and made the final editing. PH performed the electron microscopic preparations. SML reviewed and discussed the manuscript. All authors read and approved the final manuscript.
